# Pneumorrhachis in Acute Asthma Exacerbation in an 18-Year-Old Female Patient: A Report of a Rare Case

**DOI:** 10.7759/cureus.87331

**Published:** 2025-07-05

**Authors:** Omar Abdelaziz, Hashim Abid, Ado Yusuf, Nickh Uppal

**Affiliations:** 1 Internal Medicine, Manchester University National Health Service (NHS) Foundation Trust, Manchester, GBR; 2 Radiology, Manchester University National Health Service (NHS) Foundation Trust, Manchester, GBR

**Keywords:** asthma exacerbation, barotrauma, pneumomediastinum, pneumorrhachis, subcutaneous emphysema

## Abstract

Pneumorrhachis (intraspinal air) is a rare radiological finding, often associated with trauma, iatrogenic causes, or increased intrathoracic pressures. We present a case of pneumorrhachis secondary to severe asthma exacerbation, highlighting its pathophysiology and clinical implications. An 18-year-old female patient presented with a history of dry cough, chest tightness, and pain in her neck. Subsequent scans, including chest X-rays and computed tomography (CT) scans, showed a large volume pneumomediastinum and pneumorrhachis. The patient was managed conservatively with bronchodilators, corticosteroids, and supplemental oxygen, leading to the resolution of symptoms and radiographic abnormalities.

This case illustrates that pneumorrhachis can occur in asthma due to increased intrathoracic pressure, leading to air dissection into the spinal canal. Conservative management suffices in most cases, as seen here.

Pneumorrhachis should be considered in severe asthma exacerbations with pneumomediastinum, even in the absence of neurological symptoms. Early recognition and treatment of the underlying asthma are crucial to prevent complications. This case adds to the limited literature on pneumorrhachis in non-traumatic settings.

## Introduction

Pneumorrhachis (PR), the presence of intraspinal air, is an exceptional but eminent radiographic finding, accompanied by different etiologies and possible pathways of air entry into the spinal canal [1]. In the absence of trauma or iatrogenic causes, spontaneous PR has been reported secondary to respiratory tract infection, abdominal infections, asthma, vomiting, and acute cough and is usually preceded by pneumomediastinum [2]. An acute increase in the intra-alveolar pressure leads to rupture of alveoli resulting in air escape into the perivascular space. This air further moves through the facial planes into the posterior mediastinum and thus into the epidural space [3]. Spontaneous PR is extremely rare, and only a few cases have been reported in the literature [1]. There are no guidelines for the management of PR. Treatment of the inciting etiology, such as bronchodilators and oxygen supplementation for asthma exacerbation, abscess drainage, chest tube for pneumothorax, etc., is required [4].

## Case presentation

An 18-year-old female patient presented to the emergency department (ED) of a district general hospital with a dry cough and chest tightness. She also complained of pain overlying her right shoulder, jaw, and neck, which was exacerbated by deep breaths and swallowing. 

This was initially managed as an acute severe exacerbation of asthma. The patient had a past medical history of childhood asthma that was diagnosed at the age of three but without acute exacerbations for several years. She had not utilized inhalers for four years preceding the presentation due to being asymptomatic. Social history comprised a smoking history of three years, alongside cannabis use. 

On examination, chest auscultation revealed diffuse bilateral wheeze and palpable subcutaneous emphysema at the right base of the neck. Investigations showed a mild increase in inflammation markers (Table 1).

**Table 1:** Summary of key laboratory findings

Test	Result	Normal value
White blood cell (WBC) count	13.3 x 10^9^/L	4.0-11.0 x 10^9^/L
Neutrophils	9.82 x 10^9^/L	1.80-7.50 x 10^9^/L
Eosinophils	0.33 x 10^9^/L	0.00-0.40 x 10^9^/L
Potassium	3.3 mmol/L	3.5-5.0 mmol/L
Sodium	141 mmol/L	135-145 mmol/L
C-reactive protein (CRP)	58	<1
Magnesium	0.92 mmol/L	0.7-1.0 mmol/L

**Figure 1:**
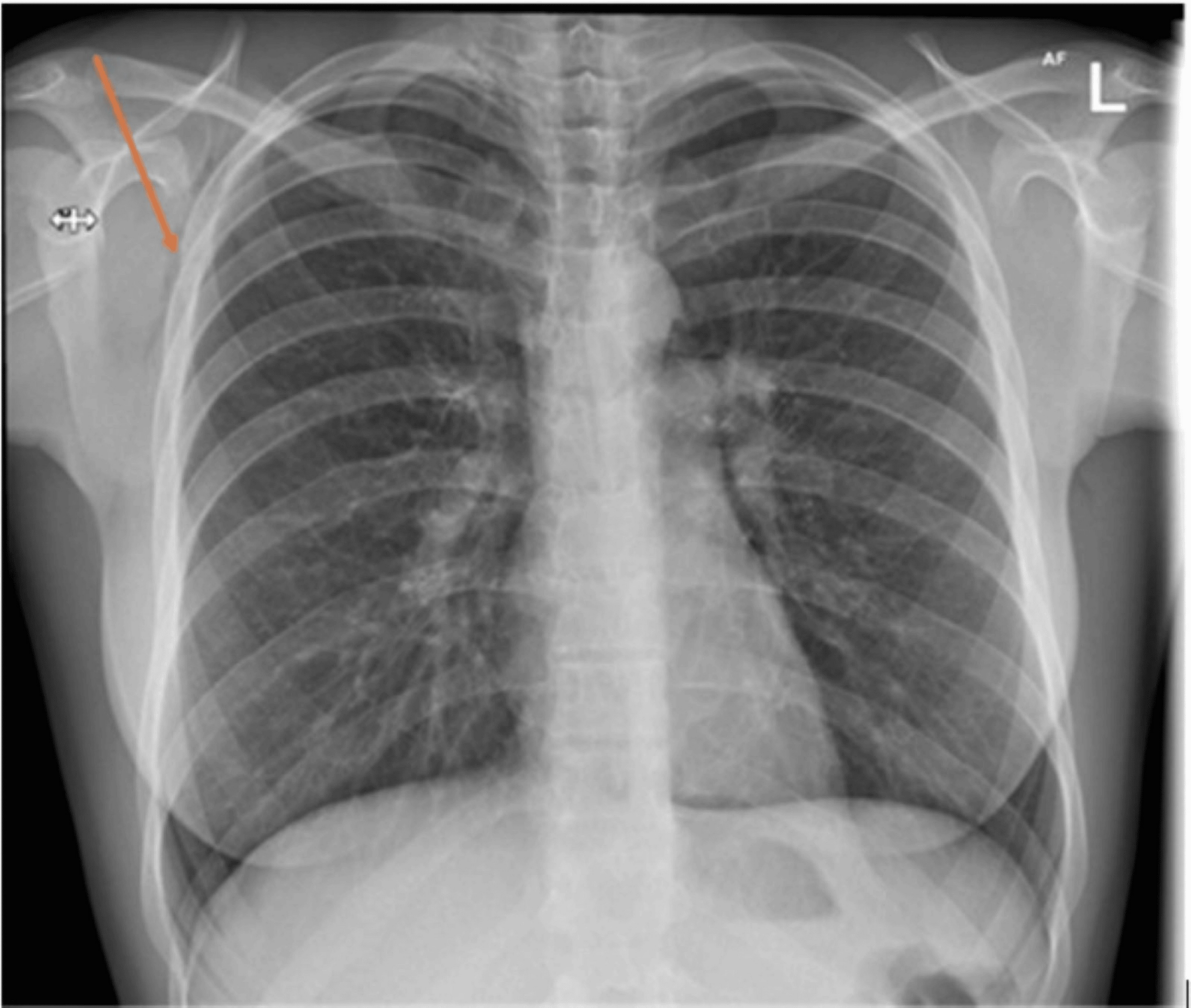
X-ray showing streaking linear lucencies overlying the superior mediastinum and surgical emphysema in the right axilla

A chest X-ray was performed (Figure 1), which showed streaking linear lucencies overlying the superior mediastinum and surgical emphysema in the right axilla. 

**Figure 2:**
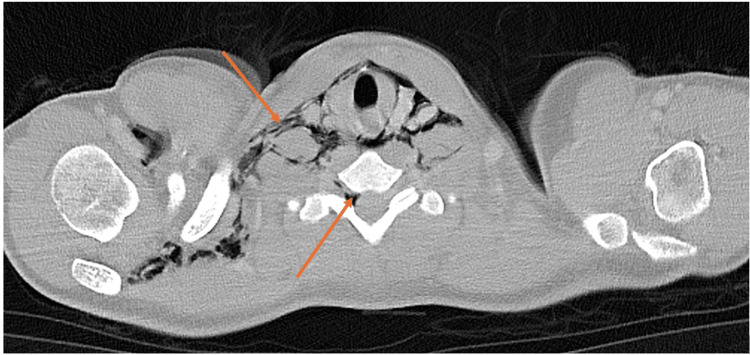
CT showing large volume pneumomediastinum with extensive surgical emphysema of the neck and right axilla/chest wall CT: computed tomography

**Figure 3:**
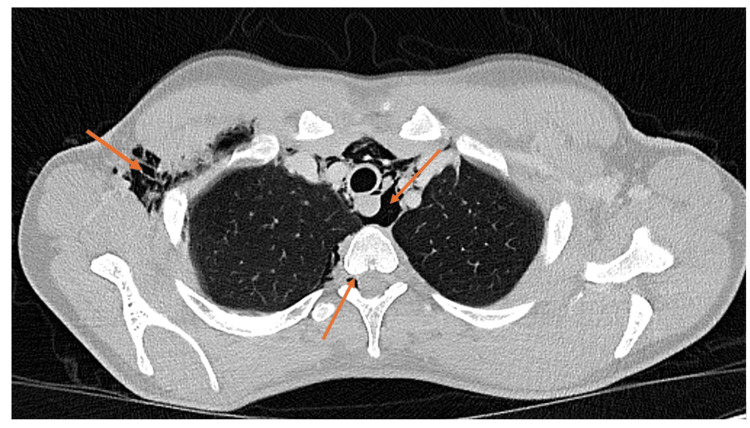
CT scan showing large volume pneumomediastinum with extensive surgical emphysema of the neck and right axilla/chest wall CT: computed tomography

Computed tomography (CT) thorax-abdomen with oral and intravenous contrast was performed to rule out an esophageal rupture (Figures 2, 3). This confirmed large volume pneumomediastinum with extensive surgical emphysema of the neck and right axilla/chest wall. Air was seen tracking into the spinal canal via the right intervertebral foramen from the deep neck fascia in the lower cervical spine, causing PR. There was a small left pneumothorax within the left oblique fissure and anteriorly. No evidence of esophageal perforation was observed. In addition to initial management for asthma exacerbation, the case was discussed with cardiothoracic surgery, who advised that as the lung was fully expanded, there was no current indication for chest drain, opting instead for conservative management with monitoring and oxygen.

At this point, the diagnosis was revised to non-traumatic spontaneous pneumomediastinum secondary to bronchial asthma. Following a review by respiratory physicians, the management consisted of regular nebulizers and steroids, oxygen supplementation with 2 L via nasal cannula, and adequate analgesia. A neurological examination was also done, which was normal.

A repeat chest X-ray was performed the day after admission due to ongoing pain, which showed a larger volume and worsening of the pneumomediastinum. Further discussion with cardiothoracic surgery was performed, who advised that there was no indication for acute surgical intervention. The case was also discussed with neurosurgery, who confirmed that no neurosurgical intervention was indicated and advised to avoid strenuous activities. Gastroenterology also advised that there was no indication for esophagogastroduodenoscopy (EGD). 

**Figure 4:**
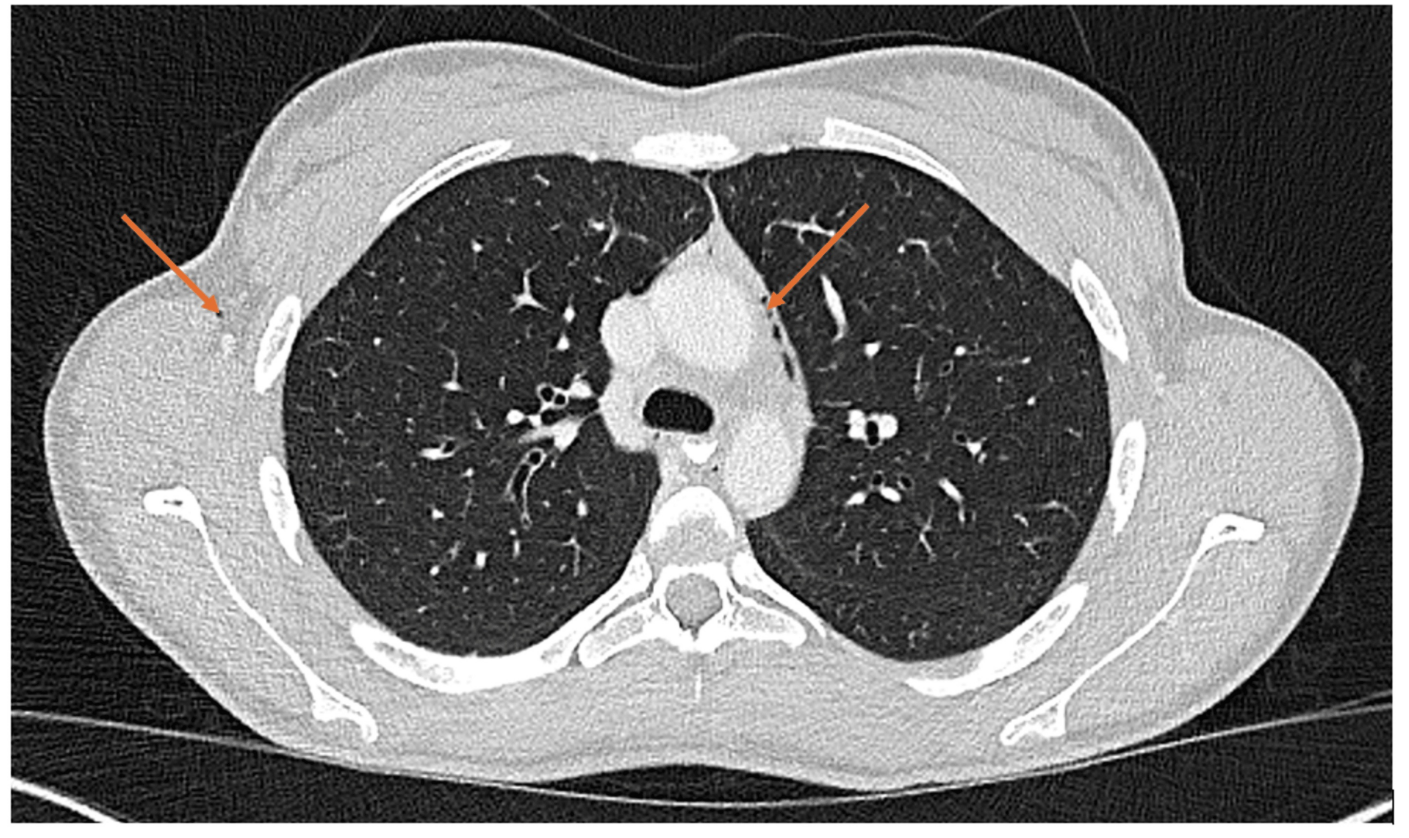
CT showing resolution of pneumorrhachis and pneumomediastinum CT: computed tomography

CT thorax-abdomen was done on the fifth day of admission (Figure 4), which showed interval resolution of the pneumothoraces and volume reduction of the surgical emphysema and pneumomediastinum. 

Following a period of supportive management and monitoring, the symptoms as described above improved. The patient was discharged after six days as an inpatient, with follow-up organized with the respiratory pleural team as an outpatient. 

She was seen in the pleural clinic a week after her discharge, with a repeat chest X-ray demonstrating normal appearances without evidence of subcutaneous emphysema, pneumothorax, or pneumomediastinum. In order to optimize her asthma control, inhaled therapy was initiated alongside a referral to the asthma team. She was discharged from the pleural clinic.

## Discussion

Pathophysiology of PR in asthma

PR is typically caused by the introduction of air into the spinal canal through various mechanisms, including trauma, iatrogenic causes, or increased intrathoracic pressure [1]. In asthma exacerbations, the forceful coughing and bronchospasm associated with severe airflow obstruction can lead to alveolar rupture, resulting in pneumomediastinum [5]. Air can also dissect in the retropharyngeal and retroperitoneal space causing discomfort and respiratory compromise. In rare situations, air can dissect between the mediastinum and the upper spine causing PR [6]. This mechanism aligns with the findings in our patient, who presented with severe coughing episodes preceding the identification of PR.

Diagnostic considerations 

CT is considered to be the diagnostic modality of choice, with a sensitivity of 100% [7]. In our case, the patient’s CT scan revealed air within the spinal canal, confirming the diagnosis. Importantly, PR is often asymptomatic, as seen in this patient, but it can occasionally present with neurological symptoms such as radiculopathy or myelopathy if the air compresses neural structures. The absence of neurological deficits in this case underscores the importance of considering PR even in asymptomatic patients with predisposing conditions.

Clinical significance and management 

The identification of PR in asthma exacerbations highlights the potential complications of increased intrathoracic pressure in severe asthma. While PR itself is often benign and self-limiting, its presence should prompt clinicians to evaluate for other complications of barotrauma, such as pneumothorax or pneumomediastinum [8]. In this case, the patient’s pneumomediastinum resolved with conservative management, including bronchodilators, corticosteroids, and oxygen therapy. High‐concentration oxygen therapy may be considered, as it may promote re‐absorption of gas into soft tissue in subcutaneous emphysema, pneumomediastinum, and presumably PR [6].

Uniqueness of the case

This case is noteworthy due to the rarity of PR in asthma exacerbation and the absence of neurological symptoms despite the presence of intraspinal air. Spontaneous pneumomediastinum is an uncommon entity primarily affecting young adults and children. There have been only a few case reports of PR associated with spontaneous pneumomediastinum [9]. 

Gupta et al. [10] described a rare case of spontaneous giant PR with symptoms that included back pain and deteriorating mobility, managed surgically with laminectomy without fusion. Gibikote et al. [9] described an even rarer case of PR in the pediatric population: a four-year-old boy with a traumatic case of PR that resolved spontaneously. Extensive extradural PR was also described by Garcia-Cebrián et al. [11], with the cause being related to obstetric analgesia. Poovazhagi et al. [12] present the case of a seven-year-old girl with accidental injury, presenting with emphysema, pneumomediastinum, pneumothorax, pneumoperitoneum, and PR. Fantacci et al. [13] reported a case of respiratory syncytial virus associated with PR, requiring rapid recognition and management. 

These cases highlight the fact that the causes of PR are many, and while it may be complicated by various conditions and associated with neurological symptoms such as meningeal irritation, extremity weakness, and cauda equina syndrome, it may also present in the asymptomatic patient. These learning points are further impressed in the report as described by ourselves, where careful consideration of differentials as well as extensive follow-up was essential.

Implications for clinical practice

As with other rare conditions, there are no published guidelines for the ED management of PR, necessitating the use of case presentations to educate providers as to the complications and plan of care of this diagnosis [14]. Early recognition and appropriate management of the underlying asthma are crucial to prevent further complications. Additionally, this case underscores the value of CT imaging in diagnosing PR and ruling out other potential causes of air leakage.

## Conclusions

In summary, PR is a rare but important complication of asthma exacerbation that can occur due to increased intrathoracic pressure and air dissection into the spinal canal. This case underscores the importance of considering atypical complications in asthma exacerbations and the need for vigilant monitoring and appropriate imaging to guide management. It also adds to the limited literature on PR in asthma and emphasizes the importance of considering and prompt management of this diagnosis in patients with severe respiratory distress.
